# The complete chloroplast genome of *Cephalotaxus lanceolata* (Taxaceae), a plant species with extremely small populations

**DOI:** 10.1080/23802359.2021.1901621

**Published:** 2021-03-19

**Authors:** Jia Ge, Yun Xiong, Guifen Luo

**Affiliations:** a Yunnan Key Laboratory for Integrative Conservation of Plant Species with Extremely Small Populations, Kunming Institute of Botany, Chinese Academy of Sciences, Kunming, P. R China; b Key Laboratory for Plant Diversity and Biogeography of East Asia, Kunming Institute of Botany, Chinese Academy of Sciences, Kunming, P. R China; c Gongshan Administrative Sub-Bureau of Gaoligongshan National Nature Reserve, Nujiang, P. R China

**Keywords:** *Cephalotaxus lanceolata*, chloroplast genome, plastid genome, plant species with extremely small populations

## Abstract

*Cephalotaxus lanceolata* K. M. Feng ex C. Y. Cheng W. C. Cheng and L. K. Fu (Taxaceae) is a threatened plant species and a typical plant species with extremely small populations (PSESP) with only four individuals found in Gaoligong Mountain. With the aim of providing data for future conservation efforts, we sequenced the whole chloroplast (cp) genome of *C. lanceolata.* The results showed that the plastid genome is 136,404 bp in size. In total, 116 unique genes were annotated, including 83 protein-coding genes, 29 tRNA genes, and 4 rRNA genes. The total GC content was 35.1%. We performed phylogenetic analyses based on 12 cp genomes of Taxaceae, and we determined that the genus *Cephalotaxus* forms a sister group to *Taxus* and *Pseudotaxus*.

The genus *Cephalotaxus* (Taxaceae) is endemic to southern and eastern Asia comprising nine species according to the Plant List (http://www.plantlist.org). Phytochemical research indicated that the constituents identified from this genus such as the cephalotaxin-type alkaloids and flavonoids were found to have remarkable anticancer activities (Chen et al. 2017). Owing to the medical value, the *Cephalotaxus* genus has been overharvesting for decades. Unfortunately, there is few conservation action or conservation biology research conduct on this genus. *Cephalotaxus lanceolata* K. M. Feng ex C. Y. Cheng W. C. Cheng and L. K. Fu is restrict distributed along the upper reaches of Dulong River in Gongshan County, northwestern Yunnan, only four individuals have been found so for. According to the literature, it is also distributed in Myanmar, but it is deficient in the distributional data. It was categorized as Endangered (EN) in the Red List (Yang and Liao 2013), and it is a typical plant species with extremely small populations (PSESP) (Sun et al. 2019). The only four *C. lanceolata* individuals need an urgent conservation attention. At this point, we report and characterized the complete plastome of *C. lanceolata* for the further conservation efforts.

In August 2020, we collected the fresh leave material of *C. lanceolata* from Gaoligong Mountain, Yunnan Province of China (27.68°N, 93.30°E). The voucher herbarium specimen was stored in the herbarium of Gongshan Administrative Sub-Bureau of Gaoligongshan National Nature Reserve (voucher: XY2020091). The complete chloroplast (cp) genome sequencing was performed on the Illumina Hiseq X platform (Illumina Inc., San Diego, CA, USA), assembled into contigs using the *de novo* assembler SPAdes version 3.11.0 (Bankevich et al. 2012) with complete plastome of *C. sinensis* (Accession no.: NC_037245.1) as the reference. Initial annotations were made by using plann 1.1 (Huang and Cronk 2015) and PGA (Qu et al. 2019). *Cephalotaxus lanceolata* cp genome was deposited in Genbank under the Accession no.: MW149080.

The complete plastome sequence of *C. lanceolata* is 136,404 bp in length. Similar to other conifers, the studied species does not contain inverted repeat regions (IRs) (Ge et al. 2019). The total GC content was 35.1%. The plastome encodes 116 genes, including 83 protein-coding genes, 29 tRNA genes, and 4 rRNA genes. Eleven genes contained a single intron (comprising five protein-coding and six tRNA genes), *clpP* and *ycf3* encoded two introns (Table S1).

To reveal the phylogenetic position of *C. lanceolata*, a phylogenetic tree was conducted based on complete plastid genomes of 12 species, from Taxaceae with *Pinus taeda* and *Picea asperata* (Pinaceae clade) as outgroups. The genome-wide alignment of all cp genomes was done by HomBlocks (Bi et al. 2018), resulting in 77,234 positions in total. The whole genome alignment was analyzed by IQ-TREE version 1.6.6 (Nguyen et al. 2015) under the TVM + F+G4 model. The tree topology was verified under both 1000 bootstrap and 1000 replicates of SH-aLRT test. Trees were visualized in Figtree version 1.4.3 (Andrew 2016). The phylogenetic tree showed that *C. lanceolata* was found to be closely related to *C. sinensis* and together form a sister group to *C. oliveri*. The genus *Cephalotaxus* forms a sister group to *Taxus* and *Pseudotaxus*, and these three genus together form a sister group to *Torreya* and *Amentotaxus* (Figure 1).

**Figure 1.**
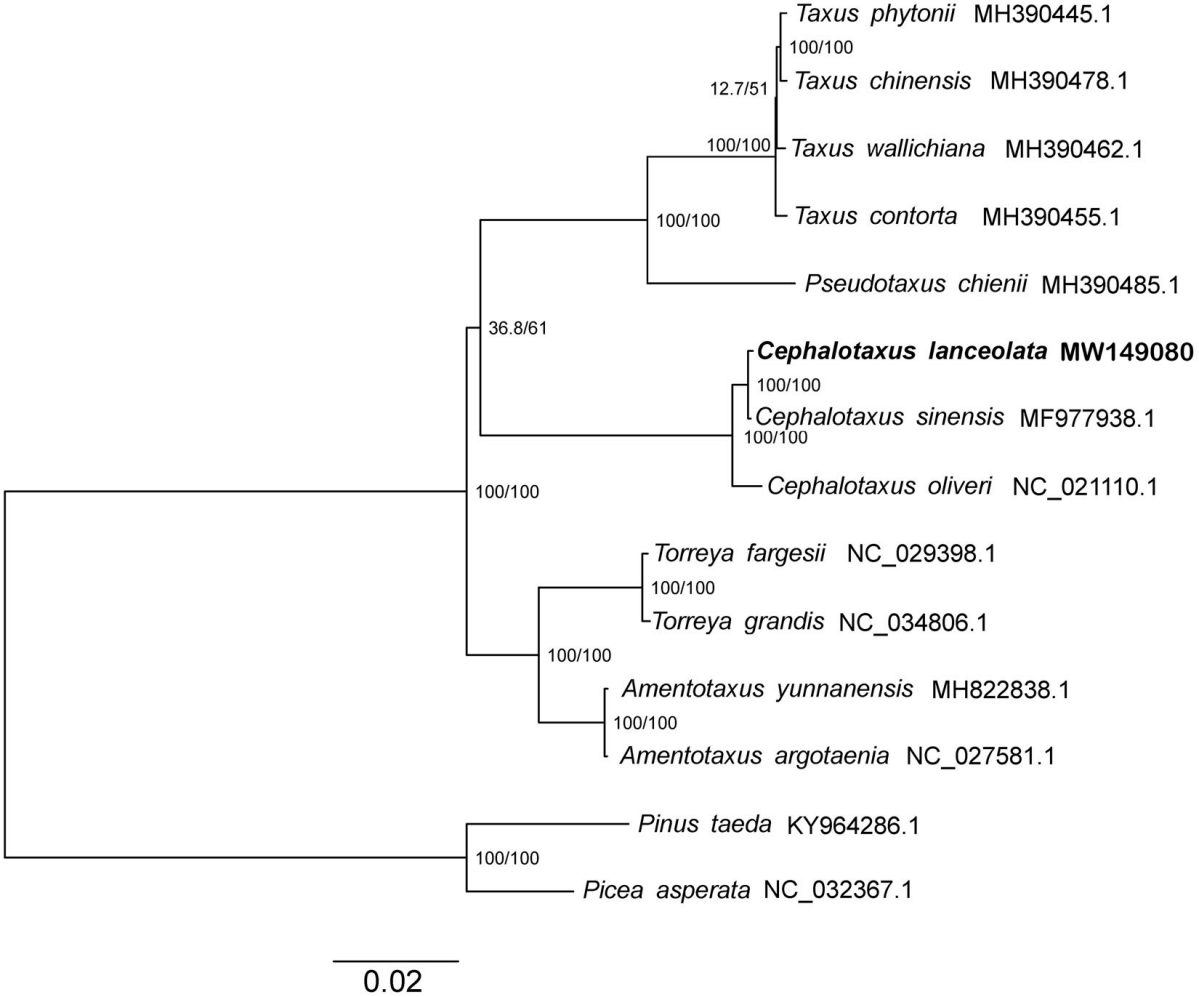
The phylogeny recovered from 12 complete chloroplast genomes of Taxaceae based on the maximum-likelihood (ML) analysis. *Pinus taeda* and *Picea asperata* were used as outgroups. The ML consensus tree is shown with bootstrap supports indicated by numbers beside the branching point.

## Data Availability

The genome sequence data that support the findings of this study are openly available in GenBank of NCBI at (https://www.ncbi.nlm.nih.gov/) under the accession no. MW149080. The associated BioProject, SRA and Bio-Sample numbers are PRJNA6852599, SRR13254579 and SAMN17080720 respectively.
